# Understanding the effect of irradiation temperature on microstructural evolution of 20MnMoNi55 steel

**DOI:** 10.1038/s41598-022-18538-5

**Published:** 2022-09-30

**Authors:** A. P. Srivastava, S. K. Sharma, S. Saini, S. Neogy, S. K. Ghosh, D. Kabiraj, R. Tewari

**Affiliations:** 1 Materials Science Division, Bhabha Atomic Research Centre, Trombay, Mumbai 400085 India; 2 Radiochemistry Division, Bhabha Atomic Research Centre, Trombay, Mumbai 400085 India; 3 MaterialsProcessing and Corrosion Engineering Division, Bhabha Atomic Research Centre, Trombay, Mumbai 400085 India; 4 Inter-University Accelerator Centre, New Delhi, 110067 India

**Keywords:** Materials science, Structural materials, Theory and computation

## Abstract

In this study, the effect of irradiation temperature on microstructural evolution of Indian RPV steel is reported. This study, by virtue of helium ion irradiation at 77, 300 and 573 K, could bring out the effect of the irradiation induced defects on microstructural and mechanical property changes at different stages of their existence starting from the state of cascade damage till the point of their free migration. Irradiation experiments were performed with varying ion energies to achieve nearly uniform irradiation damage of 0.05, 0.2 and 3 dpa in a ~ 300 nm wide region. Irradiated samples were characterized using GIXRD, PAS, TEM and nanoindentation. Unirradiated samples showed predominant presence of a combination of di- and tri-vacancy type of defects. Most of the dislocations present in unirradiated samples were screw dislocations, while mixed type was noticed upon irradiation irrespective of the irradiation temperature. PAS study showed formation of distinct defect types at different irradiation temperatures. TEM study confirmed formation of dislocation loops and defect clusters on irradiation. Higher irradiation temperatures resulted in the extension of the width of the damage region owing to increased migration of defects.

## Introduction

Reactor pressure vessel (RPV), made up of special grade low alloy steel, acts as a pressure boundary in light water reactors. During reactor operation, RPVs are known to get exposed to modest doses of neutron irradiation in their lifetime (typically ~ 0.05—0.1 dpa after 40 years of operation)^1,2^. This seemingly small dose, however, is sufficient to reduce the fracture toughness of RPV significantly^1–3^. Hence, a study on the performance of RPV steel under irradiation assumes a lot of significance. Under reactor operating conditions, when a high energy neutron enters in a structural material, it usually dislodges a host atom from its lattice position with high kinetic energy. This dislodged atom, which is commonly known as primary knock on atom (PKA), displaces other host atoms from their lattice positions creating collision cascade which generates large number of point defects such as vacancies, self-interstitial atoms (SIA) and their clusters. The concentration, configuration and distribution of these irradiation induced defects in the microstructure finally govern the structural integrity of any in-core reactor structural material^4,5^.

Radiation damage is typically quantified in terms of displacement per atom (dpa)^5^. Radiation damage imparted by neutrons and ions in a material, in general, can be compared if the dpa imparted by both the species are same^1,5^. In this regard, heavy ion irradiation can be very useful, as irradiation by heavy ions can quickly attain similar level of neutron damage in much shorter time without any radiation hazard. Similar to neutron irradiation, heavy ion irradiation also results in the formation of dense, large displacement cascades thereby generating similar configuration of vacancies, self-interstitial atoms and their clusters.

These point defects generated during irradiation tend to migrate towards defect sinks such as grain boundaries, existing dislocations and surface boundaries. They may annihilate at such sites or mutually recombine or may form larger sized clusters^4,5^. The irradiation induced defects can be efficiently characterized in terms of type, density and distribution using non-destructive techniques such as positron annihilation spectroscopy (PAS) and X-ray diffraction line profile analysis (XRDLPA)^6–11^. It is known that XRDLPA can probe larger size defect clusters and dislocation loops very well. The same technique is, however, not quite suited for detection of scattered vacancy type of defects or small size clusters as they contribute mostly towards diffuse scattering in XRD and not towards peak broadening. On the contrary, positron as a probe is very sensitive to such point defects and hence the information generated by positron is complementary to the information deduced from XRD. It should as well be noted that as the damage due to heavy ion irradiation is limited only to a few hundreds of nanometers from the surface, the nanoindentation technique can also be used to measure the change in hardness value (*H*) due to irradiation.

PAS consists of the measurements of positron lifetime as well as Doppler broadening of positron annihilation radiation (DBAR). Positron annihilation parameters viz. positron lifetime and Doppler broadening are highly sensitive to electron density at positron annihilation sites present within a material. Positrons have a high propensity to get trapped easily at the open volume defects due to absence of positively charged nuclear core in these defects. As a result, PAS is highly sensitive to open volume defects up to mono-vacancies with their number density at ppm level^12,13^. In the case of heavy ion-irradiation induced defect studies of metals and alloys, the damage or defects are confined to a very narrow sub-surface region depending on the energy of the radiation. These types of samples are not amenable for investigation using conventional PAS methods due to continuous energy distribution of positrons emitted from the radioisotope. In this regard, slow positron beam, which can deliver monoenergetic positrons of required energy, can play a significant role. Using these beams, a large number of studies on depth dependent local structure of crystalline materials have been carried out by measuring depth dependent Doppler broadening or lifetime in the samples^14,15^.

In the present study, indigenously developed RPV steel^16^ of SA 508 Grade 3 Class 1 type has been irradiated with helium ion to achieve various levels of damage including that which is usually experienced by such steels in nuclear reactors. The primary aim of this study has been to assess the microstructural stability of the RPV steel against irradiation damage to 0.05, 0.2 and 3 dpa imparted under varying irradiation temperature and to identify the underlying mechanisms leading to various responses with respect to dose and temperature variations. Irradiation temperature of 573 K has been selected as it is close to reactor operating temperature and a low irradiation temperature of 77 K has been selected as at this temperature, primary defect formation will be slightly enhanced whereas their diffusion will be substantially reduced compared to high temperature irradiation which in turn may dictate the observed difference in radiation damage responses, while an increase in temperature shall reflect the recombination and annihilation of defects due to enhancement in mobility. The microstructural stability has been assessed in terms of changes in domain size, microstrain, dislocation density, S-parameter (open volume defect density) and hardness. The defect microstructure developed under irradiation has been characterized using transmission electron microscopy (TEM).

## Experimentation

### Irradiation experiments

Samples of dimensions 10 X 10 X 2 mm from the surface of a shell forged RPV steel block in quenched and tempered condition were machined. Chemical composition (in wt.%) of the low alloy steel used in this study is shown in Table 1. The critical transition temperatures (A1 and A3) were measured by dilatometry using small cylindrical samples (*ϕ* 4 mm × 10 mm) with a heating rate of 5 °C/min. The A1 and A3 temperatures were determined as 726 and 817 °C respectively. Quenching and tempering process was carried out in a small resistance furnace. During the heat treatment, samples were austenitized at 875 °C for 5 h then air cooled (to simulate the near surface location of thick forging) and then tempered at 650 °C for 7 h. The surfaces of these samples were prepared through standard metallographic techniques using final polishing with colloidal silica solution. The choice of helium ion and its energy was made considering that damage should not be very close to the surface to avoid any interfering surface effects and the damage area should be easily accessible for microstructural characterization. In the present study helium ions of energies 200, 300 and 400 keV were employed with suitable variation of fluence to achieve broader region (~ 300 nm) of average uniform damage of 0.05 dpa, 0.2 dpa and 3 dpa respectively. The irradiation experiments were performed at Inter University Accelerator Centre (IUAC), New Delhi, using low energy charged particle of helium ions from an Electron Cyclotron Resonance (ECR) ion source. Helium ions of 200, 300 and 400 keV variable energies were used in combination with varying fluences of 0.48 × 10^15^, 1.07 × 10^15^ and 1.04 × 10^15^ ions/cm^2^, respectively, to achieve a ~ 300 nm wide relatively uniform damage distribution with ~ 0.05 dpa dose beneath ~ 500 nm of the irradiated surface. Similarly, different combinations of fluence values with the same set of energies were also employed to achieve such uniformly irradiated zones with damage levels of 0.2 dpa and 3 dpa. The beam current was set to 1 *μ*A in all the cases. The average damage rate was 6 × 10^−5^ dpa/s. Three sets of independent Irradiation experiments were performed in which the temperature of the sample’s surface was maintained to ~ 77 , 300 and 573 K respectively. Microstructural characterization and hardness measurements of such irradiated samples were carried out at room temperature.

**Table 1:** Chemical composition (in wt.%) of the low alloy steel used in this study.

C	Mn	Mo	Ni	Cr	Si	P	S	V	Al	Cu	Fe
0.16–0.20	1.20–1.50	0.40–0.55	0.50–1.00	0.15 max	0.15–0.40	0.012 max	0.008 max	0.02 max	0.01–0.04	0.04	Balance

### Grazing incidence X-ray diffraction

Grazing incidence X-ray diffraction (GIXRD) experiments were performed at a glancing angle of 1.5° with K_β_ filtered CoK_α1_ X-ray (*λ* = 1.78892 Å) in a Rigaku Smartlab rotating anode X-ray diffractometer.

Since the irradiated region of interest is limited to 500 to 800 nm from the irradiated surface, GIXRD was employed to probe the damage region. Penetration depth (*x*_α_) of X-ray for grazing angle *α *in GIXRD mode can be estimated using Eq. (1) as follows ^17,18^.

1 $${x}_{\alpha }=\frac{-\mathrm{ln}(1-G)}{\mu (\frac{1}{Sin\alpha }+\frac{1}{Sin(2\theta -\alpha )})}$$

where G is the fraction of the incident beam absorbed till a depth of *x*_α,_ *μ* is the linear attenuation coefficient of the material and 2θ is the diffraction angle. In the present study, fraction of unabsorbed X-ray has been taken as 1/e and angle 2θ has been taken as diffraction angle of the most intense peak corresponding to (110) plane. The density and the mass attenuation coefficient of iron corresponding to CoK_α1_ X-ray of wavelength 1.789 Å are 7.874 g/cc and 59.5 cm^2^/g, respectively. The penetration depth of X-ray for the grazing angles of 1.5°, 2°, 2.5° and 3.8° were found to be ~ 540, 713, 881 and 1300 nm, respectively.

### Transmission electron microscopic (TEM) investigation

Sample preparation for TEM was carried out using focussed ion beam (FIB) technique. Cross-sectional milling was performed to yield a TEM lamella so that the damage structure at a depth of ~ 500 nm from the irradiated surface can be examined. Initially a lamella of ~ 1 μm thickness and length and breadth of ~ 15 μm each was lifted out from the bulk specimen. The lamella was then further thinned using glancing incidence FIB to a thickness of < 100 nm amenable for TEM observation. JEOL 2000FX and Libra 200FE TEMs operating at 160 and 200 kV, respectively, were used for sample examination.

### Positron annihilation measurements

Positron annihilation lifetime spectroscopy (PALS) measurements on the unirradiated sample were carried out using a fast–fast coincidence spectrometer coupled to two plastic scintillation detectors placed opposite to each other. For each measurement the radioactive source (^22^Na ~ 10 μCi) sealed in a polyimide (film thickness ~ 7 μm) envelope was sandwiched between two samples and placed between the detectors. PALS spectra having more than one million counts were acquired. PALS spectrum of a reference sample (silicon crystal) was acquired to estimate the fraction of positrons annihilating in the polyimide films and source. The fraction of positron annihilation in the polyimide films as a result of backscattering from steel samples would be more as compared to silicon. The correction for this enhancement in source has been made according to the procedure mentioned in ref^14,15^.

Depth dependent Doppler broadening measurements were carried out on unirradiated and irradiated samples to characterize the depth profile of atomic level damage brought about by ion irradiation. The details of this experimental facility can be obtained elsewhere^15^. In brief, positrons emitted from a radioactive source (^22^Na ~ 25 mCi) are moderated using a tungsten moderator. These monoenergetic positrons are transported from the source end to the sample using magnetic field produced by a solenoid and a pair of Helmholtz coils. The energy of positrons is varied by floating the sample at requisite voltage. The Doppler broadening spectrum at each implantation energies are acquired using an HPGe detector (energy resolution 2.0 keV at 1332 keV of ^60^Co) placed parallel to the irradiated surface of the samples. The Doppler broadening of annihilation radiation is analyzed using line-shape parameters viz. S- and W-parameters. S-parameter is calculated as fractional area in the central region (511 ± 0.77 keV) whereas the W-parameter is calculated as the fractional area in the wing regions (2.30 keV ≤|Eγ – 511|≤ 5.76 keV) of the annihilation peak. S-E and W-E profiles have been fitted using variable energy positron fit (VEPFIT) program to evaluate the characteristic S- and W-parameter of the damaged region.

### Nano indentation measurements

Hardness measurements of unirradiated and irradiated samples were undertaken employing the nanoindentation technique with Berkovich diamond indenter (UNHT, CSM). The load (*P*) vs. indentation depth (*h*) measurement was carried out in depth control mode with depth varying between 300 and 900 nm. Ten measurements were conducted at each depth for each sample with spacing between individual indents set to 50 µm and the average value of these measurements was used for the interpretation.

## Results

**Figure 1:**
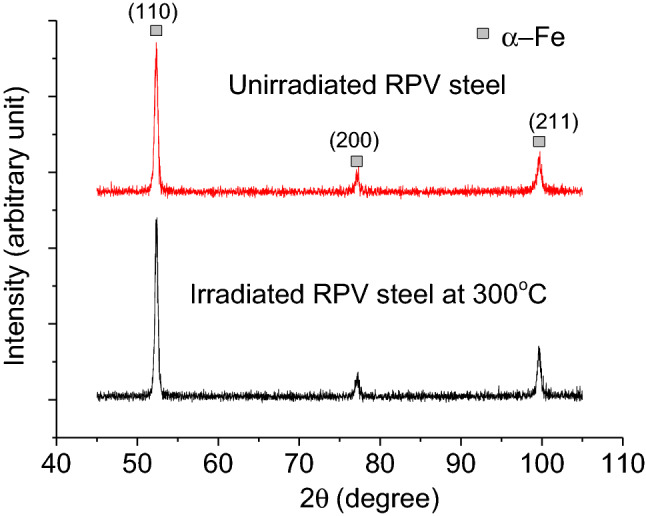
GIXRD diffractogram of unirradiated RPV steel sample and sample irradiated to 0.2 dpa at 573 K obtained at glazing angle of 2.5°.

**Figure 2:**
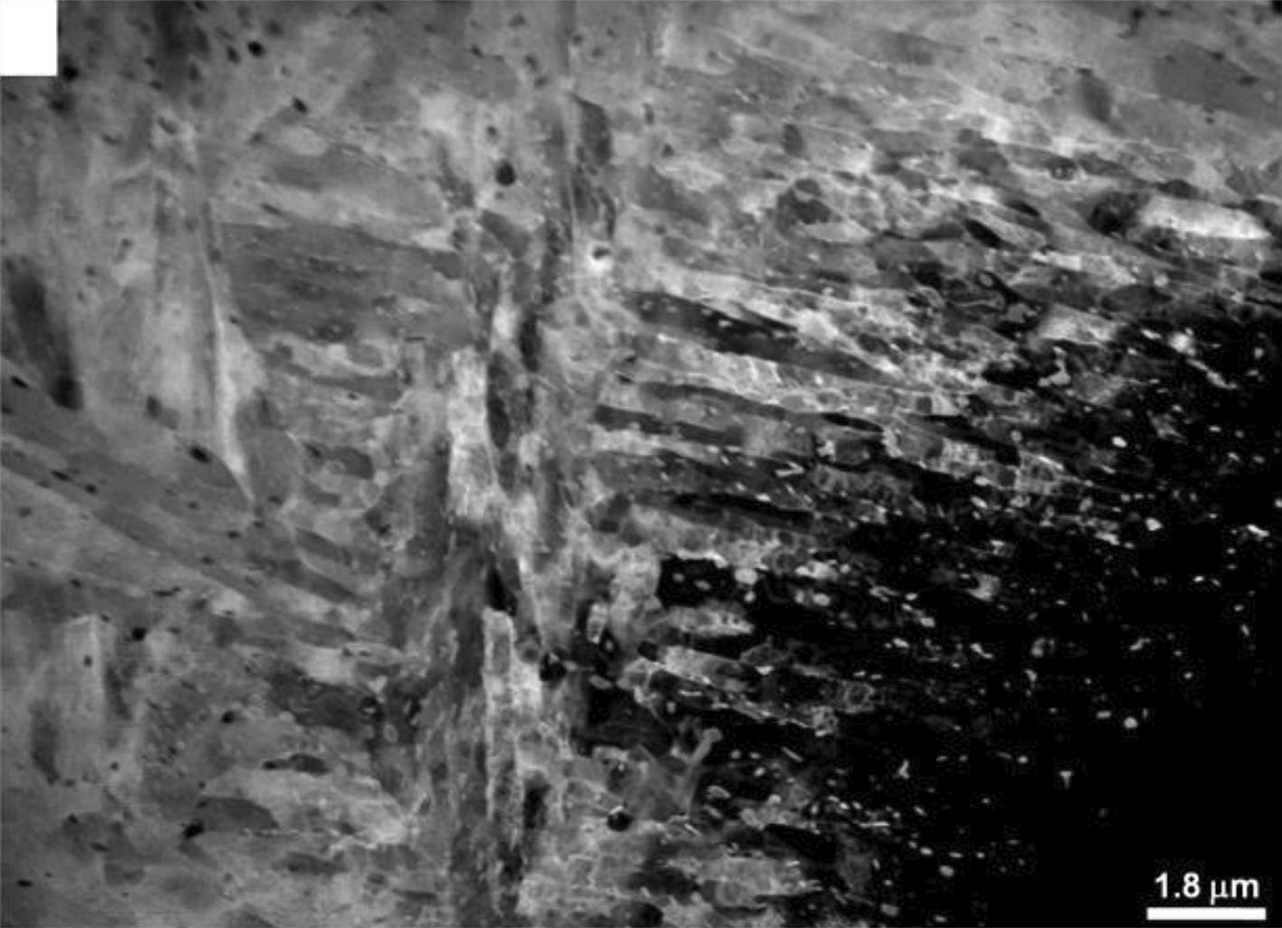
TEM micrograph showing the microstructure of RPV steel forgings in quenched and tempered condition.

The microstructure of the RPV steel in unirradiated condition was characterized using XRD and TEM. Figure 1 shows one of the grazing angle X-ray diffractogram obtained from this sample. The three diffraction peaks could be indexed to bcc α-Fe phase (Space Group: Im $\overline{3 }$ m)^19^. The lattice constant estimated from the unirradiated sample was found to be 2.87 Å. Figure 2, a representative TEM micrograph of the same sample, shows a tempered bainitic microstructure. The micrograph exhibits bainitic-ferrite laths with intra as well as intergranular cementite precipitates containing mostly Mn and appreciable fraction of Mo, as confirmed by EDS analysis.

### SRIM estimation

**Figure 3:**
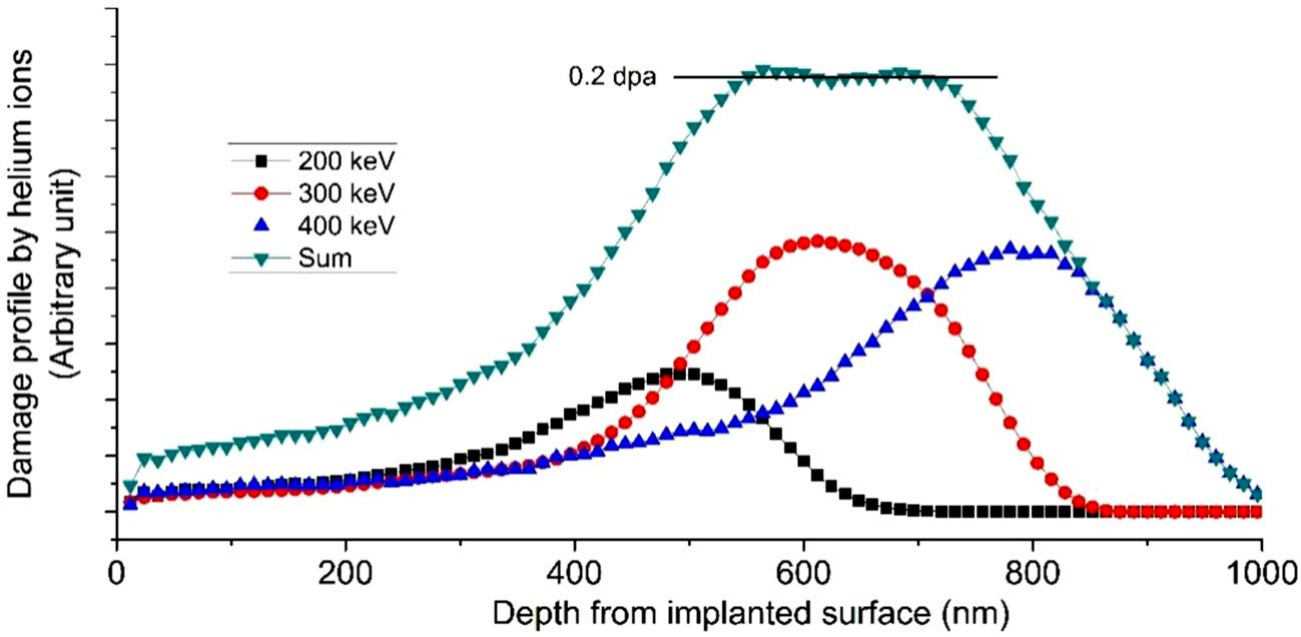
SRIM estimated damage depth profile for helium ion implantation in steel. The energies of helium ions used were 200 keV, 300 keV and 400 keV.

Estimation of radiation damage in terms of displacements per atom (dpa) and He concentration profiles for the selected set of experimental parameters was carried out with Stopping and Range of Ion in Matter (SRIM) software employing the methodology described by Stoller et. al. using NRT formula with displacement energy set as 40 eV for Fe^20^. The fluence-weighted damage profiles obtained from SRIM corresponding to these energies and their convoluted sum for 0.2 dpa average damage over the preselected region, 500 to 800 nm from the irradiated surface are shown in Fig. 3. Therefore, grazing angle of 2.5° was selected for detailed analysis, as at this particular angle the X-ray will entirely probe the damage region of interest.

### XRD study

**Figure 4:**
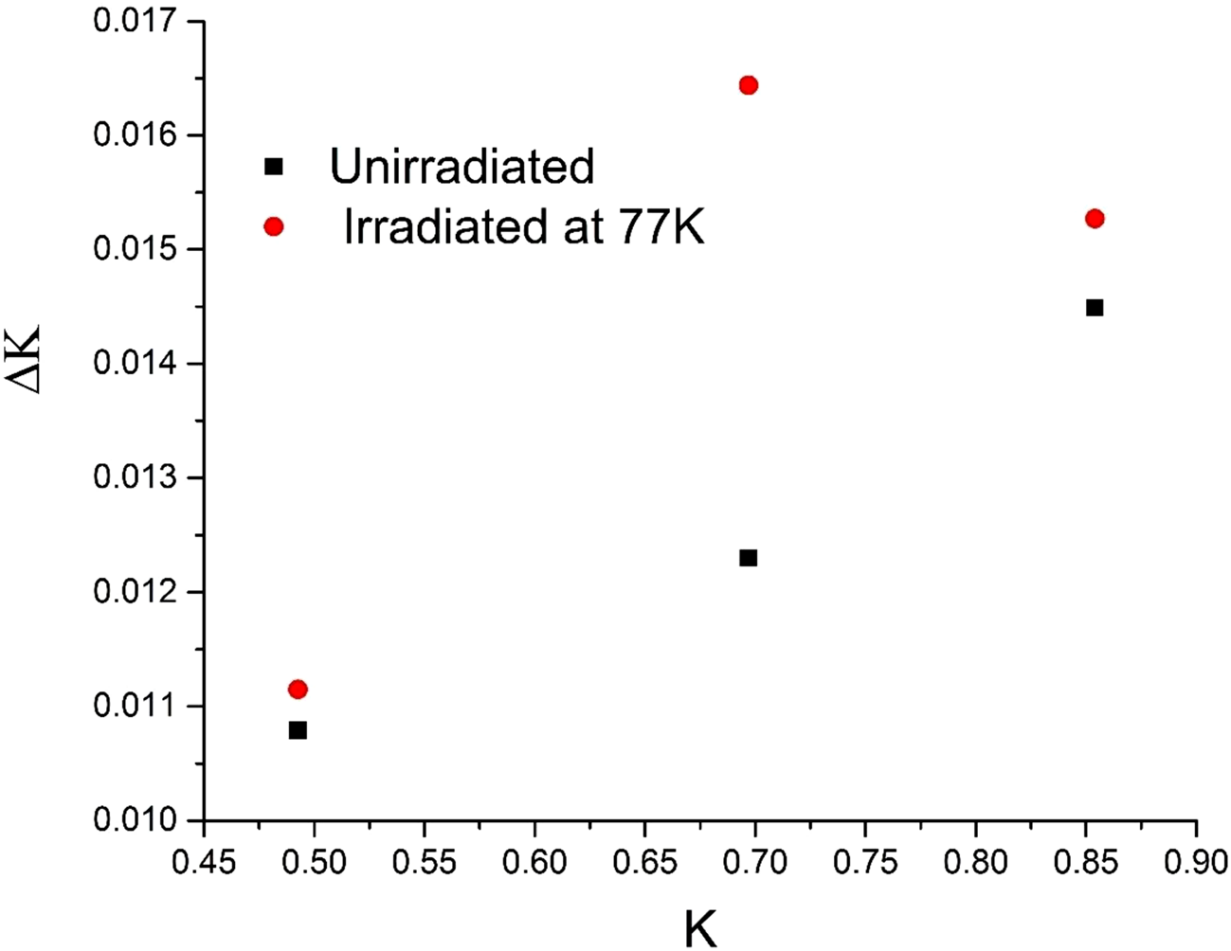
W–H plot for the unirradiated sample and for an irradiated sample at 77 K (as a representative case).

Figure 1 shows a representative diffractogram of 0.2 dpa sample irradiated at 573 K as other irradiated samples showed similar pattern. Absence of any new peak after irradiation ruled out any possibility of phase transition. To assess the irradiation response of the RPV steel, the change in the size of coherently scattering domains and microstrain due to irradiation were determined using XRDLPA, as these are known to cause broadening of the diffraction peaks. The diffraction peaks were fitted with a Lorentzian function and peak positions (2θ) and integral breadths (Δθ) were estimated. In this study, Williamson–Hall (W–H) method of XRDLPA has been applied that assumes a linear relationship between diffraction vector *K(*= *2Sin*θ*/λ)* and Δ*K (*= *2Cosθ・*Δθ/*λ)* as follows^11^:

2 $$\Delta K=\frac{1}{D}+\alpha K$$

where *D* is the domain size and *α* is twice the root mean square strain value; microstrain are supposed to originate from dislocations present in the sample. Figure 4 shows W–H plot for the unirradiated sample and for an irradiated sample at 77 K (as a representative case). It could be easily noticed that although it is possible to linearly fit the data of unirradiated sample, such a fit is not possible in the case of the irradiated sample due to scatter in the data. This indicates that strain effect due to irradiation induced dislocations is strong in irradiated samples and the strain present is no more a monotonous function of the diffraction vector K. Ungár has proposed a modified Williamson-Hall model^11^ to account this strain anisotropy by introducing an average dislocation contrast factor $\overline{C }$ in the Eq. (2) as follows:

3 $$\Delta K=\frac{1}{D}+\alpha K\surd \overline{C }$$

4 $$\overline{C }={\overline{C} }_{h00}(1-q{H}^{2})$$

5 $${H}^{2}=\frac{{h}^{2}{k}^{2}+{l}^{2}{k}^{2}{+h}^{2}{l}^{2}}{({h}^{2}+{k}^{2}+{l}^{2}{)}^{2}}$$

Here, $\overline{C }$ is the weighted average of the individual contrast factors over the permutations of the hkl indices, which is expressed as Eq. (4) for a polycrystalline cubic sample. The parameter *q* defines the character of dislocations. The parameters *q* and $\overline{C }$ both depend on the elastic constants of materials. Elastic constants for SA 508 Class 3 steel measured using resonant ultrasound spectroscopy have been reported^21^ to be *c*_*11*_ = 277.001 GPa, *c*_*12*_ = 118.715 GPa, and *c*_*44*_ = 79.143 GPa. Using these elastic constants, $\overline{C }$ for {200} reflection was determined using ANIZC program^22^ for the most common edge < 111 > {110} and < 111 > screw slip systems for a bcc material and found to be 0.2054 and 0.2222, respectively. The *q* values were calculated using the same elastic constants and the tabulated parameters for bcc structure from the work of Ungár et al.^23^ and found to be ~ 2.50 and − 0.80 for pure screw and pure edge dislocations, respectively.

**Figure 5:**
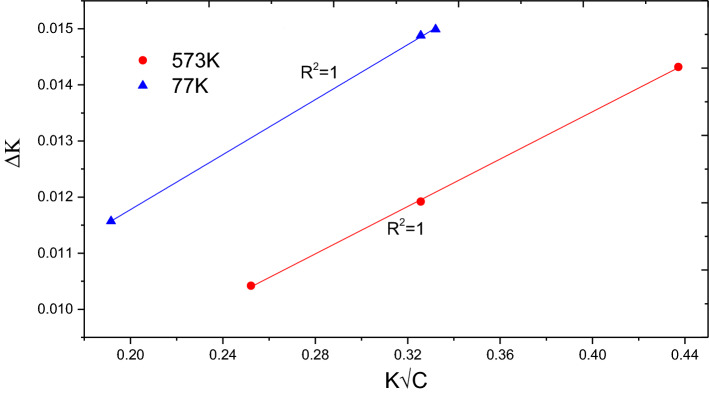
Modified W–H plot for the unirradiated sample and for samples irradiated to 0.2 dpa at varying temperatures.

By inserting Eqs. (4) and (5) into Eq. (3), a parametric linear equation with free parameters *q*, ${\overline{C} }_{h00,}$ *D* and *α* can be found, which was then solved for the experimentally observed data of *K* and Δ*K* using the method of linear regression with the constraint − 1.80 < *q* < 3.5. This constraint on ‘*q*’ was placed based on theoretically estimated *q* values with a variation of $\pm 1.$ Fig. 5 shows linearly fitted data with Eq. (4) for samples irradiated to 0.2 dpa at varying irradiation temperatures. Data from all the samples could be fitted with goodness of fit (*R*^2^) > 0.99. The fitted parameters are shown in Table 2. Since the diffraction peak broadening from microstrain is supposed to originate due to the presence of dislocations, it is possible to estimate dislocation density *ρ* using the relationship $\alpha =\surd (\frac{\pi {M}^{2}{b}^{2}}{2}\rho )$; where *b* is the modulus of the Burgers vector and the parameter *M* depends on the effective outer cut-off radius of dislocations. The value of *b* (= 2.48 Å) was estimated using the lattice constant value obtained from the XRD data. In the case of deformed samples, where large density of dislocations is present, *M* is known to vary between 1 and 2^24^. For the purpose of highlighting effect of temperature in a comparative study, *M* has been set to 1.5 for the unirradiated and 0.2 dpa irradiated samples at varying temperature and the dislocation density thus estimated is presented in Table 2.

**Table 2:** Microstructural parameters extracted from XRD data for the samples irradiated to a dose of 0.2 dpa.

Sample description	${\overline{C} }_{h00}$	q	Domain size (Å)	Microstrain ${{<\epsilon }^{2}>}^{1/2}$	*ρ* (×10^14^m^−2^)	Hardness (GPa)
Unirradiated	0.2222	3.08	185.2 ± 0.5	0.004425 ± 3.2E-5	2.01	2.34 ± 0.04
77 K	0.2183	1.23	145.4 ± 1.8	0.012248 ± 1.4E-4	15.5	3.18 ± 0.07
573 K	0.2183	−0.8	197.0 ± 4.1	0.010606 ± 1.2E-4	11.5	2.57 ± 0.03

**Figure 6:**
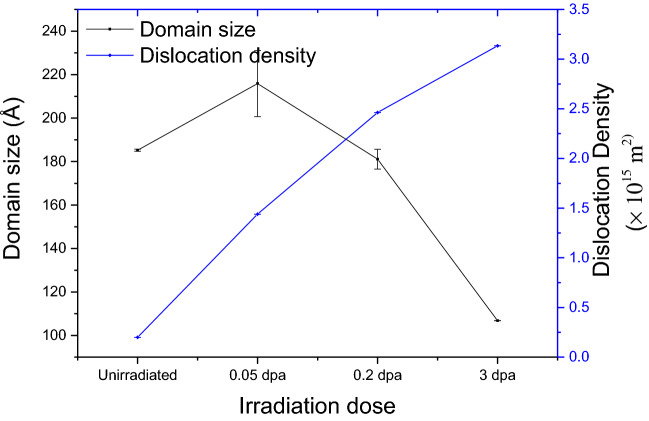
Variation of domain size with increase in irradiation dose on RPV steel samples irradiated at room temperature.

In addition to 0.2 dpa, domain size and dislocation density of samples of doses of 0.05 dpa and 3 dpa at room temperature was also carried out to bring out the effect of irradiation dose and the changes in domain size and dislocation density are shown in Fig. 6. Clearly 3 dpa sample shows maximum change in domain size and dislocation density indicating maximum damage in this sample. No saturation in defect concentration up to the investigated dose of 3 dpa is observed.

**Figure 7:**
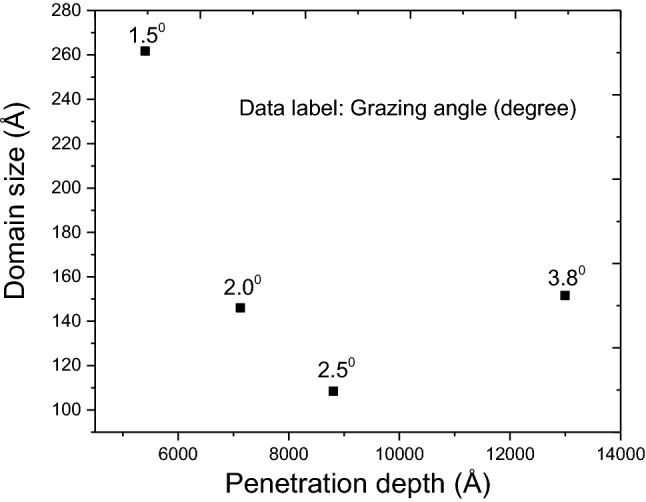
Variation of domain size as a function of implantation depth in 3 dpa sample irradiated at room temperature.

Since the sample irradiated to a dose of 3 dpa showed a pronounced change in domain size, this irradiated sample was further probed to validate the estimated depth profile from SRIM. Measured domain size at varying grazing angle for this sample has been plotted in Fig. 7. It can be clearly seen that there is a substantial dip in the domain size with depth beyond 500 nm and a minimum at around 900 nm. This damage distribution profile measured from XRD indicates that the damage zone may be extended beyond what is predicted by SRIM. This is due to the diffusion of defects that SRIM could not account for as it does not include any temperature effect in the prediction.

### Positron annihilation spectroscopy

PALS spectrum of unirradiated RPV steel samples was acquired and analyzed using PALSfit program^25^ into a sum of decaying exponentials. Two distinct positron lifetime components were noticed in the unirradiated sample. The intensity of the shorter component ($\tau_{1}$ = 104.8 ± 2.5 ps) was 65.2 ± 3.0%, while the same for the longer component ($\tau_{2}$ = 220.6 ± 5.6 ps) was 34.9 ± 2.0%. The theoretically calculated lifetime values for positron annihilation in dislocations, monovacancy, di-vacancy and tri-vacancy in pure iron are 165, 175, 197 and 232 ps, respectively ^26,27^. The bulk positron lifetime in defect free Fe is reported to be 110 ps ^28^. The presence of two positron lifetime components clearly indicates the existence of vacancy-like open volume defects in the unirradiated sample. The second lifetime value ($\tau_{2}$ ) is longer than the positron lifetime in bulk iron ($\tau_{b}$ = 110 ps). In view of theoretically estimated lifetime values, this lifetime value of 220.6 ps may be considered as an average value of positron lifetime corresponding to di- and tri-vacancy defects. It reflects that the unirradiated sample itself was having defects, predominantly a combination of di- and tri-vacancy type. This can be attributed to the thermomechanical treatments adopted in the fabrication process of these RPV steel forgings. The value of the shorter lifetime component ($\tau_{1}$) is slightly lower than $\tau_{b}$ = 110 ps. This reduction in the lifetime value can be used to estimate the density of defects using two state trapping model^26,29^. According to this model, the extent of this reduction depends on the trapping rate *K*_v_ (s^-1^) given by Eq. (6).

6 $$K_{v} = \frac{{I_{2} }}{{I_{1} }}\left( {\frac{1}{{\tau_{b} }} - \frac{1}{{\tau_{2} }}} \right)$$

7 $$K_{v} = N_{v} \mu_{v}$$

where, *N*_*v*_ is the density of defects and *μ* (s^-1^) is specific trapping coefficient. The specific trapping coefficient for monovacancies (${\mu }_{mv}$) in iron is ca. 1.1 × 10^15^ s^-1^ ^26^. The specific trapping coefficient for a multi-vacancy cluster of small radii can be obtained by multiplication of the number of vacancies in the cluster with the specific trapping coefficient for a monovacancy under the assumption that it is proportional to the number of vacancies in the cluster; this approximation is applicable in the case of clusters of small radii^29^. Thus, specific trapping coefficient in the case of sample having di and tri-vacancy defects can be taken as 2.75 × 10^15^ s^−1^. The calculated value of trapping rate *K*_v_ is found to be 2.435 × 10^9^ s^−1^. The estimated defect density using atomic number density of pure iron is ~ 7.5 × 10^22^ /m^3^.

**Figure 8:**
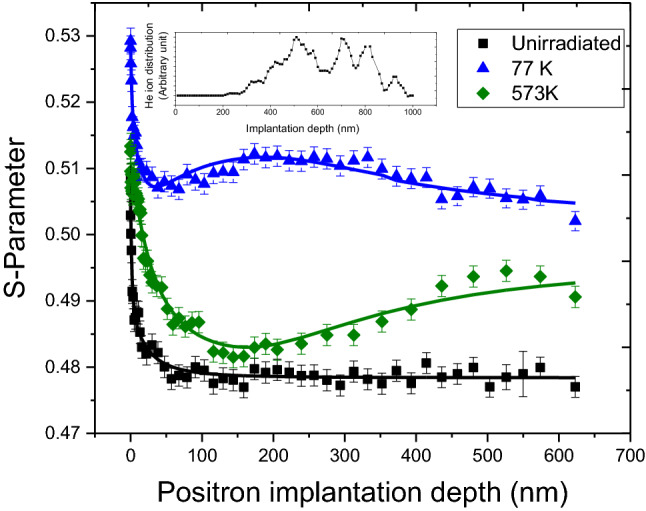
*S-E* profiles of unirradiated sample and 0.2 dpa samples irradiated at varying irradiation temperatures of 77 K and 573 K. The solid line shows the fitting of S-E profiles using the program VEPFIT.

PALS measurements do not provide any information about the spatial distribution of defects in the samples. In order to evaluate the spatial distribution of open volume defects, depth dependent Doppler broadening measurements were carried out using a slow positron beam. Figure 8 shows the *S-E* profile of unirradiated sample, where top axis of the figure shows the mean positron implantation depth < z > (in nm), which is calculated using the relation < z >  = 40E^1.6^/ρ, where E (keV) and ρ (g/cc) are the positron implantation energy and density of steel, respectively. The solid lines through the data points show the fitting of S-E profiles using the program VEPFIT. According to VEPFIT, S-parameter corresponding to the implantation energy (E, keV) can have contribution from the surface and different regions of the samples depending on the broadening of the implantation profile ^13^. In case of samples having k number of layers with different defect characteristics, S-parameter can be expressed as

8 $$S(E) = S_{{{\text{surf}}}}\, f_{{{\text{surf}}}} + \sum\limits_{i = 1}^{k} {S_{i} f_{i} }$$

where S_surf_ and S_i_ correspond to the S-parameter of the surface and *i*^*th*^ layer; *f*_*surf*_ and *f*_*i*_ represent the fraction of implanted positrons annihilating from the surface and i^th^ layer in the sample^30^.

*S-E* profile of unirradiated sample (Fig. 8) is typical of a metallic sample^8,13,30^ in which at higher implantation energy S-parameter reaches a constant value corresponding to the bulk of the sample. The higher value of S-parameter near the surface with a decrease in implantation energy or depth could be attributed to positron back diffusion to the surface where it either annihilates with the surface artifacts or forms a positronium-like state. On increasing the implantation energy, positrons are implanted deeper in the sample and their back diffusion is suppressed, leading to a decrease in the *S*-parameter. The extent in reduction of *S*-parameter as a function of positron implantation depth depends on the positron diffusion length in the sample which in turn is determined by the defect type and their number density in the sample. A single layer model with diffusion length (23.8 ± 4.2 nm) was observed sufficient to fit the S-E profile of unirradiated sample using VEPFIT. The diffusion length value observed for unirradiated sample is shorter than pure defect-free Fe^31^ indicating presence of uniform distribution of di/tri vacancy defects as confirmed from PALS measurements.

In order to investigate the effect of irradiation temperature at a fixed dose, *S-E* profiles of helium ion irradiated samples with 0.2 dpa dose at varying temperatures of 77 and 573 K are also shown in Fig. 8. The S-E profiles observed from 77 K sample which show a marginal hump at around ~ 200 nm. Presence of such hump is a deviation from the continuous increase in *S*-parameter with the implantation depth up to ~ 500 nm, as expected from damage profile obtained by SRIM calculations (Fig. 3). As per SRIM estimation most of the helium ions are deposited in the region 300–900 nm from the surface (inset of Fig. 8). He ions may form complexes ^32^ by combining with the irradiation induced vacancies that are mostly formed in 400–800 nm region. In addition, C, Mn, Ni and Mo present in the alloy may also form complexes with irradiation generated vacancies, as these elements have favorable energetics for the formation of ion-vacancy complexes^33,34^. The S-parameter for such ion-vacancy complex is lower than that for an isolated vacancy defect^32,34^. At 77 K temperature, since irradiation induced vacancies are nearly immobile, they got arrested within the region of formation i.e. 400–800 nm. This phenomenon together with deposition of He ions as well within this region (300–800 nm) resulted in increased He ion-vacancy complex formation, thereby lowering the S-value as observed in region beyond ~ 300 nm depth^32^. As the temperature of irradiation is increased to 573 K, the S-E profiles show no drop in S-parameter in the region from ~ 400–800 nm as at a higher irradiation temperature 573 K, defects formed due to irradiation have higher mobility whereas ion-vacancy complexes and vacancy clusters are unstable at higher temperature and have a tendency to dissociate into individual vacancies. All these phenomena are responsible for the observed differences in S-E profiles at 573 K in contrary to 77 K.

**Figure 9:**
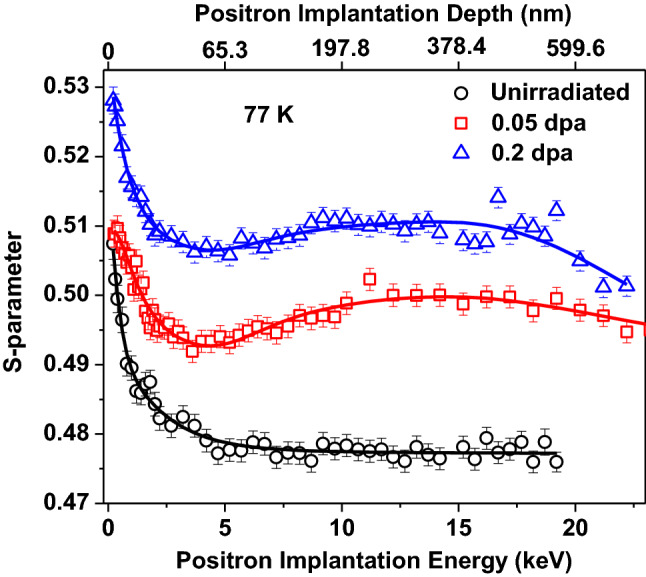
*S-E* profiles of unirradiated, and irradiated samples with peak dose of 0.05 and 0.2 dpa at 77 K. The solid lines show the fitting of the S-E profiles using VEPFIT.

**Figure 10:**
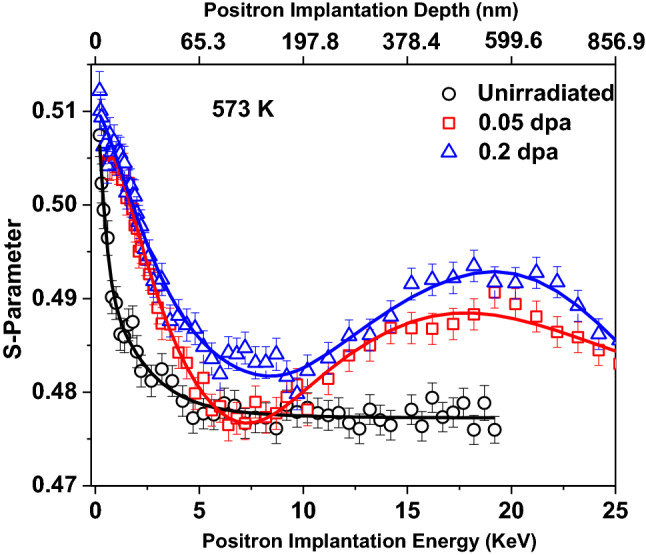
*S-E* profiles of unirradiated, and irradiated samples with peak dose of 0.05 and 0.2 dpa at 573 K. The solid lines show the fitting of the S-E profiles using VEPFIT.

Figures 9 and 10 show S-E profiles of samples irradiated at 77 and 573 K with varying dose. Clearly, density of irradiation induced open volume defects increases with the increase in dose, as a consequence S-parameter for 0.2 dpa sample remains higher than 0.05 dpa samples throughout the investigated depth of the sample. The S-E profiles of these irradiated samples are distinctly different from the S-E profile of unirradiated sample, and therefore, all could not be fit with a single layer model. This variation in S-E profiles clearly indicates a non-uniform distribution of defects in the irradiated samples, which is consistent with SRIM calculations and XRD depth profile observations (Figs. 3 and 7). A three-layer model consistent with the SRIM profile was used to fit the S-E profiles of irradiated samples at 77 K. The first two layers represent the damaged region whereas the third layer represents the non-implanted region of the sample. Preliminary fitting indicated a shorter diffusion length (< 23 nm) in the first two layers, representing the damaged region. In order to limit the number of free parameters, diffusion length of first two layers has been fixed at shorter value (10 nm) as compared to unirradiated sample and the value for the third layer has been fixed at 23 nm. The evaluated characteristic S-parameter and boundary layer of different regions are given in Table 3. The S-parameter corresponding to first layer up to ~ 125 nm (Table 3) is lower than the 2^nd^ layer for both the samples. This confirms that defect density in deeper region (beyond 125 nm to ~ 1016 nm) is higher than the first layer indicating non-uniform distribution of irradiation induced defects. The S-E profiles of 573 K samples are distinctly different from the samples irradiated at 77 K with the same doses. These S-E profiles could be fitted successfully with three-layers model. The fitting parameters for these samples are also tabulated in Table 3.

**Table 3:** Calculated S-& W-parameters and layer boundaries (BL, *nm*) using VEPFIT.

Sample/Model	S_1_	W_1_	B_L1_	S_2_	W_2_	B_L2_
Unirradiated 1 Layer	0.4772 ± 0.0003	0.0573 ± 0.0003	–	–	–	–
77 K-0.05dpa 3 Layer	0.4915 ± 0.0006	0.0495 ± 0.0006	124.6 ± 13.1	0.5007 ± 0.0006	0.0492 ± 0.0007	877.8 ± 71.7
77 K-0.2dpa 3 Layer	0.5057 ± 0.0007	0.0455 ± 0.0007	124.4 ± 22.1	0.5112 ± 0.0005	0.0469 ± 0.0005	1016.0 ± 36.7
573 K-0.05dpa 3 Layer	0.4711 ± 0.0007	0.0596 ± 0.0008	275.7 ± 11.9	0.4927 ± 0.0010	0.0485 ± 0.0009	1028.9 ± 62.6
573 K-0.2dpa 3 Layer	0.4767 ± 0.0007	0.0550 ± 0.0006	348.3 ± 17.2	0.4974 ± 0.0009	0.0473 ± 0.0010	1289.7 ± 42.3

**Figure 11:**
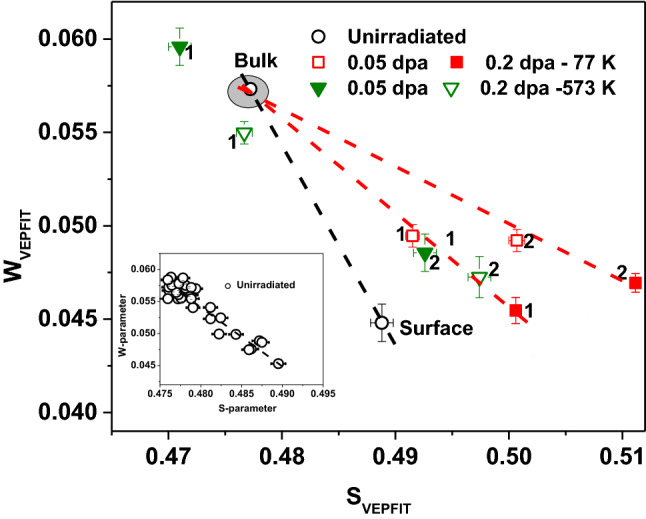
*S-W* correlation of unirradiated and irradiated samples corresponding to two different regions evaluated using VEPFIT. The inset shows the S-W correlation of unirradiated sample using experimental S- and W-parameters. Label 1 and 2 represent the 1^st^ and 2^nd^ layer starting from the surface for the fitting the S-E profiles using VEPFIT (see Table 3).

The experimental W-E profiles were fitted using VEPFIT of all the irradiated samples and unirradiated sample. Diffusion length and layer boundaries evaluated from fitting of S-E profiles have been fixed to evaluate the characteristic W-parameter of the damaged region using VEPFIT. In order to further investigate the effect of irradiation temperature and dose on type and density of defects, S-W correlations are shown in Fig. 11. The inset of the figure shows S-W correlation of unirradiated sample using experimental S- and W-parameters. The data points follow a straight line confirming the uniform distribution of same type of defect in this sample. These defects have been already identified as di/tri- vacancy defects using PALS analysis.

## TEM study

**Figure 12:**
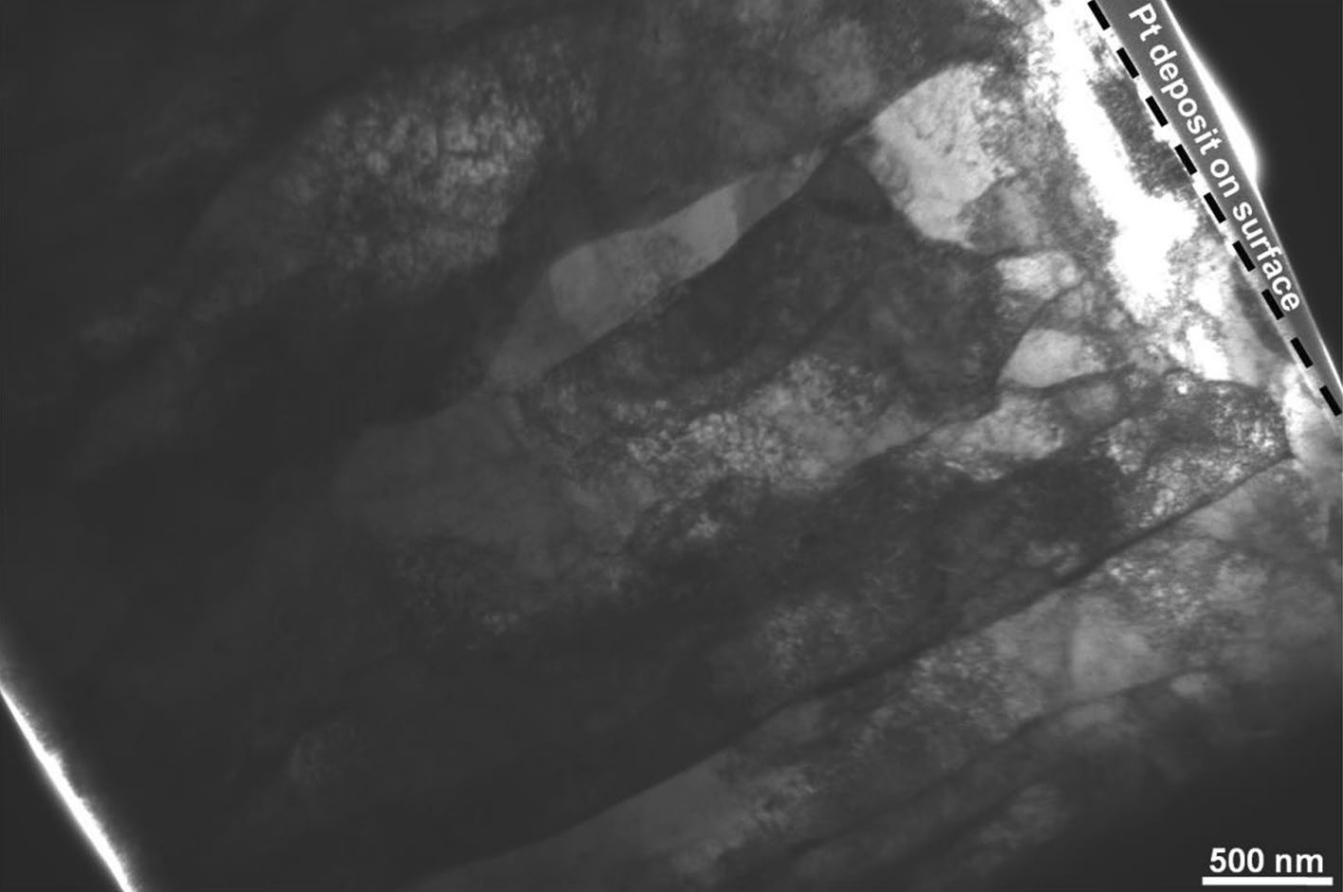
Bright-field TEM micrograph of the FIB lamella depicting the crosssection of sampleirradiated to 0.2 dpa at 77 K temperature.

**Figure 13:**
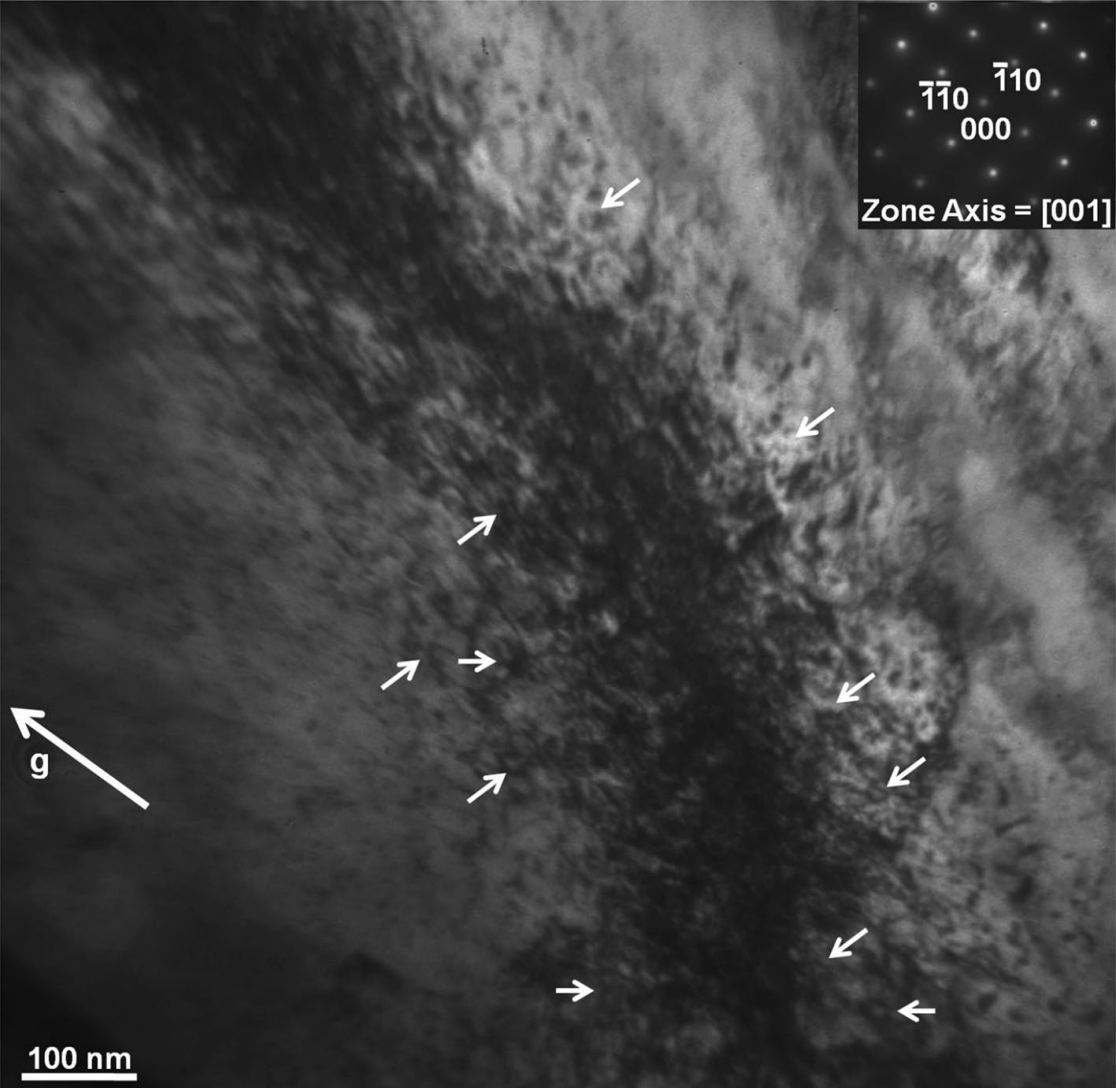
Bright-field TEM micrograph of the steel sample showing the damage structure generated upon irradiation (0.2 dpa) at 77 K temperature. The dislocation loops are marked by small arows. The diffraction conditions used for imaging are provided as inset in form of diffraction pattern and g-vector.

**Figure 14:**
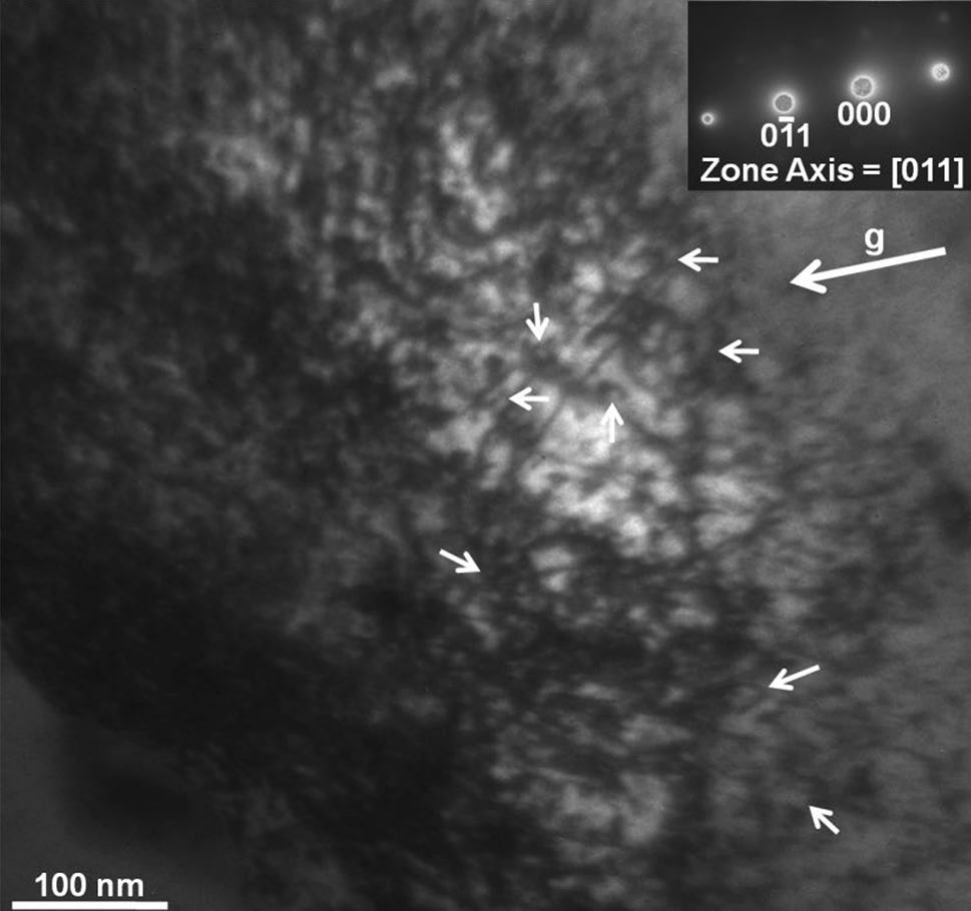
Bright-field TEM micrograph of the sampleirradiated to 0.2 dpa at 77 K temperature showing an insight within the damage zone. The dislocation loops are marked by small arows. The diffraction conditions used for imaging are provided as inset in form of diffraction pattern and g-vector.

The microstructure of samples irradiated to the fluence of 0.2 dpa at 77 K temperature was characterized using TEM. Figure 12 shows a low magnification TEM micrograph of the cross section of the sample prepared using FIB. The entire FIB-lamella is shown in the micrograph wherein the irradiated surface is marked with a dashed line. The lath-like feature corresponds to bainitic ferrite. The irradiation damage structure within a depth of 1 μm from the surface was examined under two-beam diffraction condition. Figure 13 shows a higher magnification TEM micrograph of the sample, wherein a dense irradiation damage zone of ~ 300 nm width, exhibiting dark contrast, could be observed at a depth of 500 nm from the surface. This observation corroborated the predictions of SRIM calculations presented before. Bright-field TEM micrograph in Fig. 14 shows an insight within the damage zone wherein the dislocation loops are marked by small arows. The diffraction conditions used for imaging are provided as inset in form of diffraction pattern and g-vector.

### Nanoindentation study

**Figure 15:**
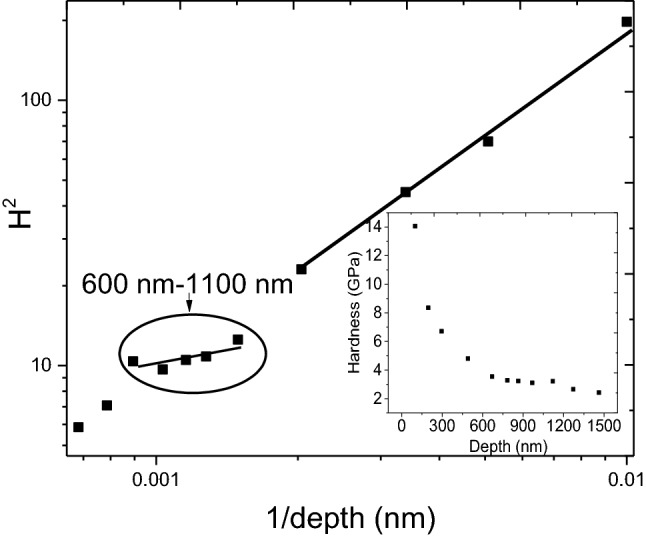
Nix-Gao plot for 3 dpa sample irradiated at room temperature. The inset shows hardness measured as a function of depth from the implanted surface.

The point defects generated due to irradiation is expected to alter the mechanical properties of the steel samples. Since the irradiation damage limited to only few hundreds of nanometers from the sample surface, the technique of nanoindentation has been used to bring out the changes in the hardness. Since measurements were made near the surface, an indentation size effect (ISE) has been accounted for using Nix-Gao model^35,36^, which can be described using the following equation:

9 $${H}^{2}={H}_{0}^{2}\left(1+\frac{{h}^{*}}{h}\right)$$

where *H* is the measured hardness at a depth *h*, h* is the characteristic depth which characterizes the depth-dependence of hardness and *H*_*0*_ is the macroscopic hardness. Hardness measured as a function of depth from the implanted surface on 3 dpa room temperature irradiated sample is shown in inset of Fig. 15. Near the surface, hardness *H* was found to decrease with an increase in depth till ~ 600 nm, and then maintaining nearly the same value up to a depth of ~ 1100 nm. Thereafter, a decrease in hardness value could be noticed due to contribution from the unirradiated part of the sample which is better known as soft substrate effect (SSE). Increase in value of *H* near the irradiated surface is due to ISE effect, and to account for the same, plot of *H*^*2*^ vs *1/h* shown in the same figure was analysed in view of Eq. (9). Clearly three distinct regions characterized by three different slopes can be seen. The first region is from the implanted surface up to a depth of ~ 500 nm, second region extend from 600 to 1100 nm followed by 1100 nm and beyond. This hardness profile complements the observation from XRD damage profile (Fig. 7) and PAS study (Table 3) which also suggest damage to be extended slightly more than that predicted by SRIM estimation (~ 1000 nm).

## Discussion

By combining the results of various experimental techniques employed in this work, the effect of temperature on the response of studied RPV steel to He ion irradiation can be elucidated which could be used to understand the underlying mechanism responsible for the observed changes in the microstructure and mechanical properties. Firstly, it is clear from XRDLPA analysis that the irradiated samples showed (Table 2) one order higher dislocation density in comparison to unirradiated sample which is a clear indication of the creation of additional dislocations by irradiation. A substantial increase in dislocation density even at 77 K (which is lower than the temperature required for defect migration) suggested formation of dislocation loops within the displacement cascade itself by the process of in-cascade clustering. It was further observed that the unirradiated sample (Table 1) mainly contains screw type of dislocations (with ${\overline{C} }_{h00}=0.2222$ and *q* = 3.08). Thus, the measured hardening or the strain contribution to diffraction peaks in this sample is mostly due to the dominant presence of screw dislocations. In fact, screw dislocations are known to dictate mechanical properties in the RPV steel which has bcc crystal structure^37–39^. In contrast to unirradiated sample, irradiated samples showed *q* values which could be associated with mixed dislocations (edge and screw) in the sample irradiated at 77 K (*q* = 1.23) and edge dislocations in the sample irradiated at 573 K (*q* =  − 0.8). Clearly, irradiation irrespective of temperature of experiment, increases the number density of edge component. This is expected because dislocation loops generated under the effect of irradiation, in general, assume edge character which has also been clearly brought out by Lehtinen et al.^40^ and B.C. Masters^41^ in irradiated bcc Fe.

In the sample irradiated at 77 K, where mobility of defects (vacancies, SIAs) is insignificant, smallest domain size is observed (Table 2) by virtue of the reduction in recombination and annihilation of the irradiation induced point defects at sinks at such a low temperature. This led to increased survival of point defects, defect clusters and these clusters collapsed to form dislocation loops which got manifested in increased dislocation density. This would also result in correspondingly smaller coherently scattering regions i.e. smaller domain size. Whereas, in case of sample irradiated at 573 K, an increase in the size of domain, even with respect to the unirradiated sample, clearly indicated an ongoing recovery process by virtue of annihilation of pre-existing defects with newly formed irradiation induced defects due to increased mobility of the latter. This is also reflected in decrease in dislocation density with an increase in irradiation temperature. In the process of recovery, the screw dislocations which have high degree of freedom for movement, with the help of additional irradiation induced defects, can migrate easily. In contrast, due to the requirement of well-defined slip system by the edge dislocations their motion will be restricted. This will lead to more annihilation of screw dislocations (the pre-existing ones) in comparison of newly formed edge dislocations. Therefore, sample irradiated at 573 K shows dominating presence of edge dislocations.

This effect of temperature is also evident from the *S-E* profiles obtained from the sample irradiated at 77 K (Fig. 8), which shows a higher increase in the *S*-parameter throughout the investigated depth in comparison to the sample irradiated at 573 K, indicating the presence of more open volume defects in the sample irradiated at 77 K. This decreasing trend of S value with increasing temperature resembled the changes observed in domain size and dislocation density, as estimated from the XRD study.

In order to probe the effect of temperature on the migration of irradiation induced defects deeper, boundary layers from VEPFIT can be considered (Table 3). The boundary of second layer at 77 K for 0.05 and 0.2 dpa samples were estimated to be at ~ 877 and ~ 1016 nm, respectively. These values match closely with the values predicted from the SRIM calculations, which show that the damaged region is extended up to ~ 1000 nm in the irradiated samples. However, with increase in temperature as well as dose, these boundaries of first and second layers were observed to move to deeper depths (Table 3). It indicates that the defects created at higher temperature are highly mobile and diffuses through the lattice. In case of sample irradiated at 573 K, contrary to the sample irradiated at 77 K, the diffusion length for the first layer is longer (~ 37.3 nm for 0.05 dpa and 46.3 nm for 0.2 dpa) than bulk diffusion length (~ 23 nm) for unirradiated sample. Longer diffusion length is an indication of presence of lesser defects in this layer of the irradiated sample than in the unirradiated sample. These observations are consistent with XRDPLA wherein annihilation of preexisting defects has been observed as a result of annihilation with ion-irradiation induced defects in 573 K samples.

S-W plot of irradiated sample (Fig. 11) provides insight on the type of defects created due to irradiation. The type of defects in case of unirradiated sample has been already discussed using positron lifetime spectroscopy. In case of irradiated samples, S-W data points corresponding to two different layers within the damaged region (using VEPFIT) do not fall on the connecting line of S-W data points of surface and bulk of the unirradiated sample. This deviation indicates that irradiation has changed the nature of open volume defects and these are distinctly different from the unirradiated sample. The S-W data points corresponding to 1st and 2nd layer of sample irradiated at 77 K do not follow a single trend line confirming the presence of different types of defects in each region. This difference could be attributed to the presence of irradiating species (He) predominantly in the 2nd layer, wherein it may form complex with the open volume defects in the 2nd layer as discussed earlier. The data points corresponding to 1st and 2nd layers at two different doses (0.05 and 0.2 dpa) for sample irradiated at 77 K follow their own trend lines respectively. It shows that though the density of defects in each layer increases with increasing dose from 0.05 to 0.2 dpa, the type of defects in each regions remains same. On raising the irradiation temperature to 573 K, He-vacancy complex formation probability reduces which is confirmed by the observation that the S-W data points corresponding to 2nd layer of these samples follow the trend line of the 1st layer of samples irradiated at 77 K. This also suggests that type of defects created at 573 K remain similar to that at 77 K-layer 1, where they do not form the He-vacancy complexes. In case of 573 K samples, the S-W data point location of the 1st layer (0.05 and 0.2 dpa) shows S-parameter lower than the unirradiated sample, clearly indicating the annealing of the defects in this region as the high temperature facilitated migration of defects (also manifested as longer positron diffusion length, as mentioned above). This is in agreement with the observation of higher domain size in these samples, compared to unirradiated one.

As discussed earlier, the effect of irradiation dose at a fixed irradiation temperature can be highlighted from Fig. 6. A small increase in domain size at 0.05 dpa compared to unirradiated sample may be attributed to annihilation of pre-existing defects with newly formed irradiation induced defects. It can be seen that as the irradiation dose increases, domain size reduces and dislocation density increases, thereby reflecting the generation of more defects at a higher dose. This observation is complementary to the observation of increase in S-parameter value with the increase in dose from S-E curve (Figs. 9 and 10), as discussed earlier.

The newly formed defects due to irradiation can be seen from the TEM micrograph of the damage region in the 77 K irradiated sample (Fig. 13). The damage zone consisted of dislocations loops and point defect clusters formed during irradiation, as already inferred from XRD and PAS studies. The damage zone was so densely populated with defect clusters and loops that the identification of these individual features became difficult. The small black dots in the micrograph represent defect clusters, whereas, the loops have been marked by small arrows. Figure 14 gives a further insight within the damage zone revealing close association of loops and defect clusters. A quantitative analysis in respect of type (vacancy and/or interstitial) and number density, however, could not be performed due to their high densities and considerable overlap. This sort of a damage structure is expected in heavy ion irradiation which is known to produce dense displacement cascades alike neutron irradiation. The concentration and spatial distribution of freely migrating point defects, surviving cascade quench, are known to govern the post irradiation microstructural evolution. Defects of similar nature (vacancy or interstitial) cluster together, and the collapse of these clusters leads to the formation of dislocation loops resulting in relaxation of matrix strain. A fraction of these defects is also lost to fixed sinks in the microstructure, like dislocations, grain boundaries, and phase boundaries apart from mutual recombination. However, irradiation at 77 K temperature impeded the diffusion of point defects which not only precluded their losses by above mechanisms, but also concentrated them over a narrow region as observed. Thus, it may be argued that the defect clusters and dislocation loops present in the 77 K irradiated sample are outcome of in-cascade clustering of irradiation induced defects. The sample irradiated at 77 K was selected as a representative of irradiated samples for electron microscopy in this study. Detailed TEM study of irradiated samples, incorporating dose and temperature variations, being the subject matter of an independent study will be reported separately.

**Figure 16:**
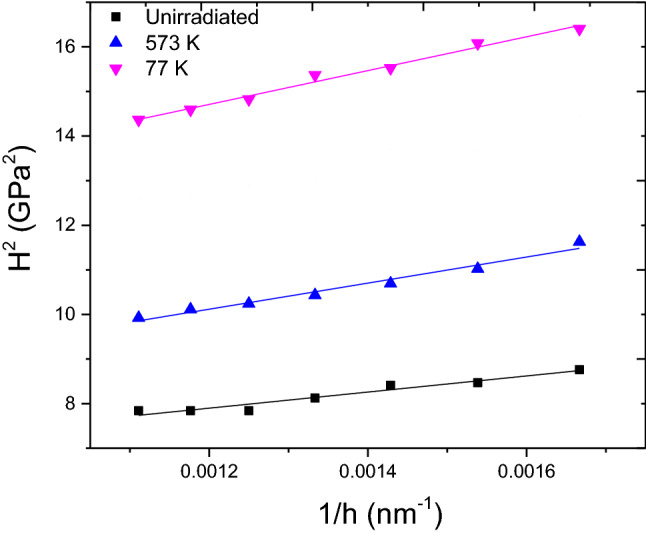
Nix-Gao plot of nanohardness for samples irradiated to 0.2 dpa at 77 K and 573 K temperature.

Presence of these radiation induced defects is expected to alter the hardness of the steel sample. In order to bring out the effect of temperature, hardness data from 600 to 900 nm of all the samples irradiated to a dose of 0.2 dpa at varying temperature were fitted linearly to Eq. (9), as shown in Fig. 16. The measured macroscopic hardness *H*_*0*_ has been given in Table 2. The irradiated samples showed increased hardness values when compared to the unirradiated sample. This is expected as the motion of dislocations is hindered by irradiation produced defects, the presence of which has been confirmed from XRD, PAS and TEM studies. In the irradiated samples, the hardness was found to decrease with an increase in irradiation temperature in agreement with the observed changes in dislocation density, domain size and S-parameter. This is reflected in lower value of domain size, higher dislocations density and higher value of S-parameter in the 77 K sample which was attributed to lower rate of annihilation of irradiation induced defects. With an increase in irradiation temperature to 573 K, hardness decreases following expected change in accordance with domain size, S-parameter and dislocation density changes due to increased mobility of defects at high temperature.

After irradiation a substantial increase in hardness was noticed in 77 K irradiated sample. In order to comprehend this change in hardness, 77 K sample has been examined in detail. The change in hardness value after irradiation, Δ*H* is 0.84 GPa. TEM image of sample irradiated at 77 K (Fig. 15) to a dose of 0.2 dpa shows the presence of small sized defect clusters and dislocation loops. Low temperature irradiation is known to cause evolution of such small sized defect clusters/loops and presence of these causes the observed radiation hardening. This change in Δ*H* has been used to roughly estimate an average defect density of these clusters and loops under the well-known dispersed barrier hardening (DBH) model which predicts the increase in yield strength caused by arbitrary defects impeding dislocation motion with defect density N, and diameter d using Eq. (10)^42–44^

10 $$\Delta {\sigma }_{y}=M\alpha \mu b\surd Nd$$

where *M* is Taylor factor (3.06 for BCC), *α* is defect barrier strength (~ 0.2), *μ* is the shear modulus (~ 79 GPa) and *b* is the magnitude of Burgers vector. The yield strength was approximated using the well-known Tabor’s empirical relationship $\Delta H\cong 3.06\Delta {\sigma }_{y}$ for BCC materials as suggested by Busby et al.^42,43,45^. Taking average defect size to be ~ 2 nm, defect density turned out to be ~ 2.57 × 10^23^ /m^3^. Assuming all the hardness contribution is from dislocations, dislocation line density *ρ*(= *N.d* in Eq. 10) was found to be 5.14 × 10^14^ /m^2^, which is close to the change in dislocation density due to irradiation in this sample observed using XRD study. The difference observed in the estimated values of dislocation density from XRD and nanohardness measurements is expected due to various approximations used.

## Conclusion

In this study, helium ion irradiation experiments were performed on indigenously developed 20MnMoNi55 grade RPV steel samples to achieve nearly uniform irradiation damage of 0.05, 0.2 and 3 dpa in a ~ 300 nm wide region lying at a depth of 500 to 800 nm beneath the irradiated surface. The observed microstructural and hardness changes due to irradiation could be rationalized in terms of defects produced in course of irradiation. TEM examination of unirradiated sample showed lath-like microstructural feature corresponding to bainitic ferrite. TEM images of irradiated sample showed an intense irradiation damage zone of ~ 300 nm width at a depth of ~ 500 nm from the surface where the formation of irradiation induced point defect clusters and dislocation loops could be seen. XRD and TEM studies confirmed that no irradiation induced phase transformation took place in samples up to 3 dpa. PAS study confirmed the presence of predominantly a combination of di- and tri-vacancy type of defects in unirradiated sample. The estimated defect density was found to be ~ 7.5 × 10^22^/m^3^. The dislocation density in unirradiated sample estimated through XRD was found to be 2.1 × 10^14^/m^2^. X-ray diffraction line profile analysis of irradiated samples showed strong strain effect caused due to irradiation induced dislocations. Nature of dislocation in unirradiated sample was found to be screw type which upon irradiation, irrespective of irradiation temperature, changed to mixed type of dislocations by favouring the formation of edge dislocations. Sample irradiated at 77 K showed lowest domain size and highest S-parameter value which could be ascribed to lower annihilation of irradiation induced defects. The dislocations and large sized defect clusters governed the observed irradiation induced hardening. The irradiation induced defect cluster/dislocation density was found to decrease with irradiation temperature. Up to the investigated dose of 3 dpa, no saturation in defect concentration was observed. PAS study indicated the formation of He-vacancy complex, which was favoured at low irradiation temperature of 77 K. This is due to the extremely low solubility of He in the metal matrix. Observations from S-parameter and domain size measurements were found to be complementary. This study clearly revealed the increased migration of defects at higher irradiation temperature. Nanohardness measurements were carried out taking into account the indentation size effect (ISE). Irradiation induced hardening was found to be maximum in 77 K sample owing to higher concentration of defects at this temperature. With increase in temperature, hardness measurement was found to follow the similar trend as of dislocation density evaluated from XRD measurements.

## Data Availability

The datasets used and/or analysed during the current study are available from the corresponding author on reasonable request.
